# Patient-Perceived Factors Influencing Physical Activity Sensor Use in Stroke Prevention and Rehabilitation: Systematic Review of Qualitative Studies Using Thematic Synthesis

**DOI:** 10.2196/86915

**Published:** 2026-03-12

**Authors:** Paul Harris, Ingrid Maine

**Affiliations:** 1 Department of Medical Education, The University of Melbourne, Grattan Street, Parkville, Melbourne, Victoria, 3010, Australia, 61 0401755482; 2 Division of Chronic and Complex Care, Western Health, St Albans, Melbourne, Victoria, Australia

**Keywords:** sensors, stroke, physical activity, rehabilitation, qualitative research

## Abstract

**Background:**

A robust correlation exists between physical activity (PA) and stroke risk reduction, and wearable PA sensors have emerged as promising adjuncts for rehabilitation and risk self-management. However, evidence regarding their long-term efficacy in facilitating sustained behavioral change remains equivocal.

**Objective:**

This study aimed to explore the barriers and facilitators influencing the effective use of PA sensors in older adults at risk of primary or recurrent stroke.

**Methods:**

A systematic search and thematic synthesis of qualitative research was conducted, focusing on PA sensor use among older adults at risk of primary or recurrent stroke. Given the emerging qualitative evidence base, inclusion was extended to proxy populations with analogous cardiovascular risk and functional profiles. Data were analyzed using line-by-line coding of primary text to generate descriptive themes and synthesize overarching analytical constructs.

**Results:**

A search of 6 bibliographic databases (January 2010 to December 2023) identified 18 eligible studies. This systematic review and thematic synthesis revealed predominantly technological (user experience and device attributes) and psychological (motivation) barriers. Key facilitators were psychological (feedback and motivation), technological (user experience), and social/environmental supports. Higher-level analysis revealed a critical interrelationship between effective user engagement and optimally assistive device characteristics.

**Conclusions:**

This review revealed a synergistic user-device interaction driving sustained PA in older adults at risk of primary or recurrent stroke. Future interventions developed in collaboration with patients and informed by the factors identified by this study will improve participation in rehabilitation and functional outcomes in this population.

## Introduction

The terms “physical activity” (PA) and “exercise,” although often used interchangeably, are distinct concepts. PA broadly refers to any bodily movement resulting in energy expenditure, while exercise is more precisely defined as a subcomponent of PA that is “planned, structured, and... done to improve or maintain one or more components of physical fitness” [1]. Within this context, physical fitness encompasses the attributes and abilities an individual possesses or acquires to perform PA or exercise. Physical inactivity, or sedentary behavior, is a significant global risk factor for mortality associated with numerous adverse health outcomes including stroke [2].

As with other cardiovascular diseases, the pathogenesis of stroke is influenced by a range of risk factors, broadly categorized into nonmodifiable, medically modifiable, and lifestyle-modifiable attributes [2]. Nonmodifiable factors include inherent characteristics such as age and genetic predisposition. Medically modifiable factors are amenable to pharmacological or surgical interventions. Lifestyle-modifiable (behavioral) factors are those that can be altered through changes to daily habits and practices. Hypertension, for example, the most significant modifiable risk factor for stroke, can be effectively managed through both medical and lifestyle interventions, including pharmacotherapy, PA, dietary changes, and weight management. Despite the established efficacy of these interventions, one study reported that fewer than 50% of patients had their stroke or cardiovascular disease risk factors appropriately assessed, treated, or managed [3]. Similarly, a recent audit of stroke rehabilitation services in Australia revealed that a comparable proportion of patients received information on self-management strategies or support programs [3]. PA is a critical, accessible, and cost-effective means of mitigating underlying risk factors, such as hypertension, contributing to the primary prevention of initial stroke events and the secondary prevention of recurrent stroke [4,5].

A growing body of evidence suggests that increased PA confers substantial health benefits across all age groups [6]. Notably, reduced sedentary time demonstrably lowers health risks and mortality, even when accounting for activity levels and established risk factors. Regular PA also mitigates the progression of hypertension and has been linked to improved weight management, reduced adiposity, and a decreased incidence of type 2 diabetes mellitus—all major risk factors for stroke. While increased PA significantly improves walking speed and balance, the evidence regarding the impact on poststroke mortality, dependence, and long-term disability remains insufficient or unclear [7]. A survey conducted in the United States revealed that a significant majority (71%) of older adults, 65 years and older, engage in self-monitoring for health indicators such as weight, diet, or exercise [8]. However, despite the prevalence of digital health tools, only a small minority (12%) utilize PA sensors and companion mobile apps. The predominant method for tracking health measures in this group was pen-and-paper [8]. The limited adoption of technology for health monitoring among this demographic may be attributable to a combination of objective factors such as PA sensor accuracy and subjective considerations such as perceived usability, which may influence engagement levels among older adults.

The need for increasingly sophisticated and persuasive strategies to motivate sustained healthy behavior remains a central challenge for clinicians designing interventions and engineers developing wearable PA sensors. Remarkably, few qualitative studies have thoroughly investigated the use of PA sensors in older adults, specifically exploring reasons for nonadoption and discontinuation. A deeper understanding of these factors is essential for the effective design and implementation of PA sensor-based interventions for stroke prevention and rehabilitation [9]. With that in mind, the current study aimed to explore the barriers and facilitators influencing the effective use of PA sensors in older adults at risk of primary or recurrent stroke.

## Methods

### Methodological Framework

This systematic review and thematic synthesis was conducted and reported according to PRISMA (Preferred Reporting Items for Systematic Reviews and Meta-Analyses) guidelines (Checklist 1) and the JBI (Joanna Briggs Institute) framework for evidence synthesis. Study quality was evaluated using JBI critical appraisal tools. Review methods were established a priori, as detailed in the published protocol [10]. The inclusion of proxy groups, such as individuals with hypertension or age-related motor deficits, is justified by their functional and psychological alignment with stroke survivors. These cohorts experience near-identical ergonomic barriers, such as the manual dexterity required for small device interfaces, and share psychological burdens, including diminished self-efficacy due to device failure, for example. By prioritizing the phenomenology of functional experience over narrow neurological diagnoses, this study applies conceptual transferability to enhance thematic depth where disease-specific evidence is still emerging [11-13]. The primary objective of the study was to identify and synthesize patient-perceived factors acting as either barriers or facilitators, influencing the use of wearable PA sensors in the context of stroke prevention and rehabilitation for older adults.

### Inclusion and Exclusion Criteria

Peer-reviewed articles published in English between January 1, 2010, and December 31, 2023, that had a primary focus on wearable PA sensor use were included in the analysis. Studies were required to have a qualitative design or a significant qualitative component. The target population included older stroke survivors and populations with analogous clinical profiles. Eligible participants were characterized by shared cardiovascular risks (eg, atrial fibrillation, hypertension), metabolic conditions (eg, type 2 diabetes, dyslipidemia), and lifestyle-related factors. Inclusion was further defined by stroke-related functional deficits, specifically, impaired physical mobility (gait and balance), cognitive decline (executive dysfunction), and reduced independence in activities of daily living [14]. Study participants were stratified into 3 age cohorts to reflect distinct clinical and life-stage profiles. The 50‐ to 64-year age group (“Young-Old”) was included to capture the critical transition period where stroke risk doubles, and this allowed for an analysis of whether PA sensor–based interventions performed differently in individuals with higher neuroplasticity and higher work/life stress [2,15]. The 65‐ to 84-year group (“Old-Old”) represents the standard geriatric stroke demographic. Finally, those aged 85+ years (“Oldest-Old”) were analyzed separately to ensure the unique frailty and comorbid complexities of the oldest-old did not skew the results of the younger cohorts.

### Search Strategy

The methodology for this synthesis followed the prepublished protocol [10]. A high-sensitivity search strategy was conducted across MEDLINE, EMBASE, CINAHL, Cochrane, PsycINFO, and Scopus using MeSH and keywords related to wearable PA sensors and exercise. To maximize recall within an emerging qualitative evidence base, population-specific filters (eg, “stroke”) were omitted; instead, the search was broadened to include all studies of populations with analogous risk and functional profiles. These results were supplemented by manual bibliography scans of included studies. This approach ensured a comprehensive capture of qualitative data despite the paucity of stroke-specific literature.

### Screening and Data Extraction

Following the prepublished protocol, publication titles and abstracts were independently screened by two reviewers (IM and PH), with a pilot test conducted to ensure interrater reliability. Disagreements were resolved through consensus or third-party adjudication. Full-text screening and deduplication were managed via Covidence. A standardized form was used to extract study characteristics (eg, demographics, research design, intervention specifics) and participant data, which were subsequently managed in NVivo. Methodological quality was evaluated using CASP (Critical Appraisal Skills Program) and JBI checklists [16,17]. Quality assessment focused on congruity of perspective, research questions or objectives, methodology, bias, participant voice, ethical standards, analysis, and conclusions. Papers were also evaluated for analytical richness, reflecting qualitative depth and interpretation with respect to the study aims.

### Data Analysis

This systematic review and thematic synthesis was conducted in 3 stages, summarized below, and discussed in more detail in the study protocol [10].

- *Stage 1. Inductive coding of included studies*: Reviewers independently coded the included studies, identifying salient sections and discussing coded sections as a team. A set of a priori codes based on the Person-Environment-Occupation-Performance and Technology Acceptance Model provided a provisional structure, with themes differentiated according to valence under binary (barrier and facilitator) top-level nodes [18,19].
- *Stage 2. Summarize under descriptive themes*: Related codes were aggregated into broader “descriptive themes” that summarized concepts across studies without going beyond the source texts. These higher-level descriptive themes were generated, discussed, and refined iteratively. Coded region frequencies (coding densities) and referenced study counts were analyzed to identify major and minor themes. The use of density metrics is well established in qualitative research, somewhat like matrix-coding queries, and is indicative of theme recurrence for included papers only [20,21].
- *Stage 3. Generate analytical constructs*: Descriptive themes were analyzed in relation to the research questions and objectives. Links between descriptive themes were mapped to generate analytical constructs and visualized diagrammatically to develop a coherent understanding from the evidence base.

## Results

### Study Selection and Characteristics

**Figure 1.**
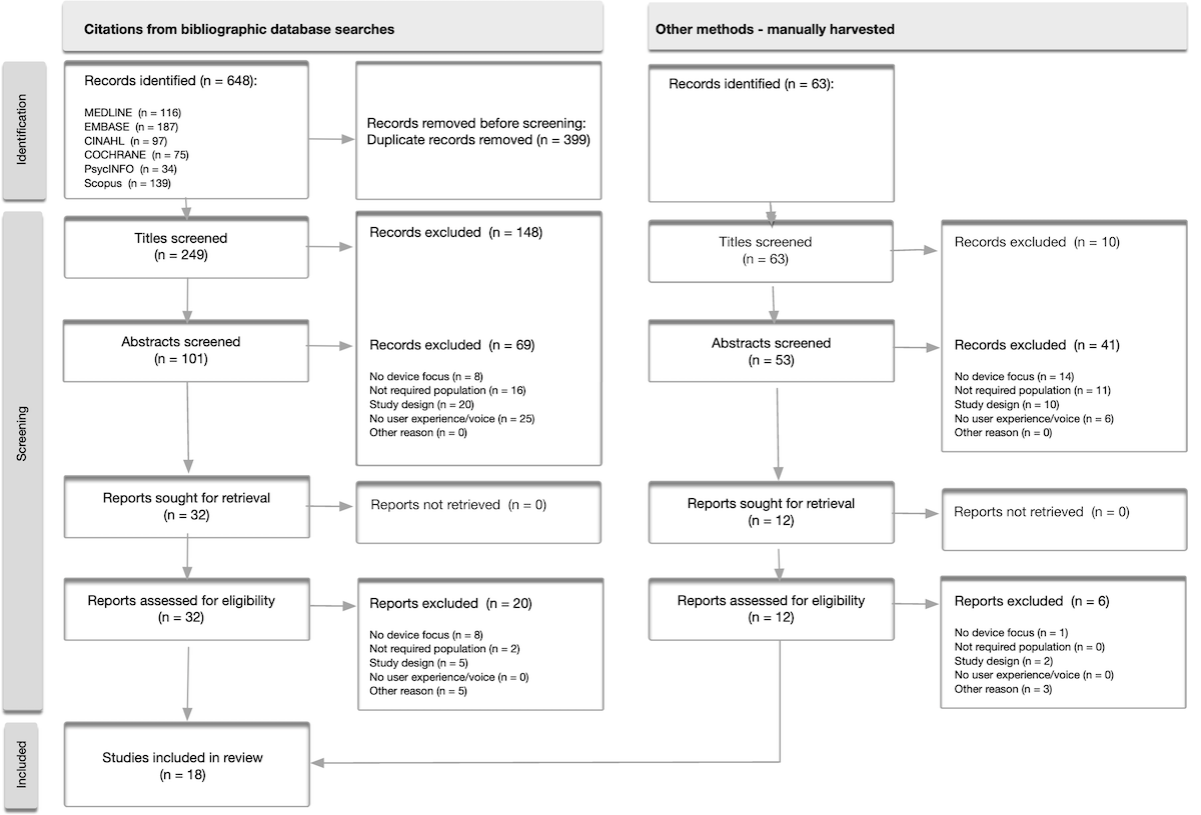
PRISMA (Preferred Reporting Items for Systematic Reviews and Meta-Analyses) flow diagram for systematic reviews.

A systematic search of 6 electronic bibliographic databases yielded 648 records. An additional 63 records were identified through manual harvesting from other methods (ie, screening citation lists of included studies). After duplicate removal and screening, 44 studies were sought for full-text retrieval. Finally, 18 studies met the inclusion criteria and were included in this systematic review and thematic synthesis. The selection process and search results are summarized in the PRISMA flow diagram (Figure 1).

To categorize heterogeneous participant data across the 18 studies listed in Table 1, a standardized coding system was applied:

- Population (N): [PRX] proxy; [STR] stroke
- Age: [YNG] 50-64; [OLD] 65-84; [ELD] ≥85 years
- Risk factors: [LFS] lifestyle; [MET] metabolic; [VAS] vascular
- Functional deficits: [ADL] activities of daily living; [COG] cognitive; [MOT] motor

**Table 1.** Characteristics of included studies.

Studies	Year	Location	Design	Duration	Pop (n)	Age group	Risk profile	Functional profile	Device(s)
Batsis et al [22]	2016	USA^a^	QLT^b^	1 mo	PRX^c^ (8)	[OLD^d^]	[LFS^e^, MET^f^, VAS^g^]	[ADL^h^, MOT^i^]	Fitbit
Nguyen et al [23]	2017	AUS^j^	QLT	18 wk	PRX (14)	[YNG^k^, OLD]	[LFS, MET, VAS]	[ADL, MOT]	Various
Schlomann [24]	2017	DEU^l^	QLT	1 y	PRX (6)	[YNG, OLD]	[LFS, MET, VAS]	[MOT]	ViFit connect
Hamilton et al [25]	2018	AUS	QLT	6 mo	STR^m^ (15)	[YNG, OLD, ELD^n^]	[LFS, MET, VAS]	[ADL, COG^o^, MOT]	Fitbit
Johansson et al [26]	2018	SWE^p^	SYS^q^, MIX^r^	NR^s^	STR (~43) PRX (54)	[YNG, OLD, ELD]	NR	[ADL, COG, MOT]	Various
Farina et al [27]	2019	GBR^t^	QLT	1 mo	STR (1) PRX (28)	[OLD, ELD]	[LFS, VAS]	[ADL, COG, MOT]	GENEactiv
Kononova et al [28]	2019	USA	QLT	6 wk	PRX (48)	[OLD, ELD]	[LFS, MET, VAS]	[ADL, MOT]	Various
Western et al [29]	2019	GBR	QLT	1 wk	PRX (31)	[YNG, OLD]	[LFS, MET, VAS]	[MOT]	SenseWear Pro
Whelan et al [30]	2019	GBR	MIX	6 wk	PRX (45)	[YNG, OLD]	[LFS, MET, VAS]	[ADL, MOT]	Fitbit, Freestyle
Støve and Larson [31]	2019	DNK^u^	QLT	3 wk	STR (20) PRX (5)	[YNG, OLD]	[LFS, VAS]	[ADL, COG, MOT]	Garmin
Ummels et al [32]	2019	NLD^v^	QLT	2‐8 wk	PRX (30)	[YNG, OLD]	[LFS, MET, VAS]	[ADL, MOT]	Tracker
Östlind et al [33]	2022	SWE	QLT	12 wk	PRX (18)	[YNG, OLD]	[LFS, MET, VAS]	[MOT]	Fitbit
Gualtieri et al [34]	2016	USA	QLT	14 wk	PRX (10)	[YNG, OLD]	[LFS, MET, VAS]	[ADL, MOT]	Withings Pulse
Mercer et al [35]	2016	CAN^w^	MIX	3+ d	STR (1) PRX (33)	[YNG, OLD]	[LFS, MET, VAS]	[ADL, MOT]	Various
Randriambelonoro et al [36]	2017	CHE^x^	QLT	7 mo	PRX (18)	[YNG, OLD]	[LFS, MET, VAS]	[ADL, MOT]	Fitbit
Ehn et al [37]	2018	SWE	QLT	9 d	PRX (8)	[OLD, ELD]	[VAS, MET, LFS]	[MOT, ADL]	Activite, Jawbone
Takemoto et al [38]	2018	USA	QLT	3 wk	PRX (15)	[YNG, OLD, ELD]	[VAS, MET, LFS]	[MOT, ADL]	Various
Brickwood et al [39]	2020	AUS	MIX	1 y	PRX (20)	[OLD]	[VAS, MET, LFS]	[MOT, ADL]	Jawbone

a USA: United States.

b QLT: qualitative.

c PRX: proxy.

d OLD: Old-Old (65–84 y).

e LFS: lifestyle.

f MET: metabolic.

g VAS: vascular.

h ADL: activities of daily living.

i MOT: motor.

j AUS: Australia.

k YNG: Young-Old (50–64 y).

l DEU: Germany.

m STR: stroke survivor.

n ELD: Oldest-Old (≥85 y).

o COG: cognitive.

p SWE: Sweden.

q SYS: systematic review.

r MIX: mixed methods.

s NR: not reported.

t GBR: Great Britain.

u DNK: Denmark.

v NLD: Netherlands.

w CAN: Canada.

x CHE: Switzerland.

Of the included studies, 1 was a systematic review that included quantitative and qualitative studies, 3 employed mixed methods, and 14 were qualitative. Studies were conducted in North America (n=5), Australia (n=3), Great Britain (n=3), and Europe (n=7). A detailed breakdown of the quality and risk of bias assessment for each study is provided in Table S1 in Multimedia Appendix 1. The characteristics of the included studies with country designations according to International Organization for Standardization 3-letter standards (eg, DEU: Germany and CHE: Switzerland) are summarized in Table 1.

Approximately 78% (n=14) of the studies included individuals with metabolic risks like obesity, prediabetes, or type 2 diabetes. Participants with vascular risks, such as hypertension, heart disease, and high cholesterol, were even more common, appearing in 83% (n=15) of the studies. All 18 studies included participants with lifestyle-related risks, specifically high sedentary behavior and poor dietary choices. Functional deficits further defined these populations: 89% (n=16) of studies reported on participants with motor or physical limitations such as joint pain from osteoarthritis, reduced walking speed, or the use of mobility aids like walkers. Participants with cognitive deficits (eg, memory loss or forgetfulness, impaired task comprehension, and execution) were represented in 4 (22%) studies, and participants with impaired activities of daily living affecting self-care and mobility were represented in 6 (33%) studies.

Specific stroke-related representation was documented in 5 studies. Johansson et al [26] systematically reviewed 24 studies of stroke survivors, while Støve and Larsen [31] included patients with stroke (80%) in active inpatient rehabilitation with hemiparesis. Similarly, Hamilton et al [25] reported a sample where 60% had a neurological primary diagnosis, including stroke, alongside significant motor deficits. Finally, Mercer et al [35] included a participant with stroke-related gait asymmetry (shuffling) that impacted activity sensor accuracy, and Farina et al [27] included a participant with stroke-related vascular dementia but no acute motor symptoms.

### Synthesis of Findings

The thematic coding frame in Table 2 was applied to all included studies and reviewed for consistency of interpretation. This process, known as “axial coding,” ensured coded themes remained connected to and understood within the context of the source studies [13].

**Table 2.** Low-level theme and subtheme nodes.

Theme	Subtheme
Built environment and technology	Acceptance, accuracy, attitudes, community, customizable, damage, familiarity, functionality, home, influences, privacy, reliability, safety, usability, usefulness, wearability
Cognitive	Attention, awareness, comprehension, language, memory, planning
Natural environment	Safety, terrain, weather
Neurobehavioral	Balance, gait, motor, sensory, vision
Physiological	Endurance, fatigue, flexibility, injury, mobility, strength
Psychological	Accountability, adherence, attainment, autonomy, competence, feedback, goals, habits, mood, motivation, preconceptions, privacy, self-awareness, self-efficacy, trust, well-being
Social support	Caregiver, information, peer, professional, training
Societal and economic	Attitudes, commitments, cost, cultural, norms, strata
Spiritual	Fulfillment, meaningfulness, values

Coding densities informed the generation of the higher-level descriptive (aggregate) themes and subthemes listed in Table 3. Density cutoff points were used to classify major (n≥30), minor (n=10‐29), and negligible (n<10) themes. Finally, descriptive themes were analyzed with respect to the research questions and objectives of the current study.

**Table 3.** Descriptive themes and subthemes.

Theme	Subtheme
Technological	User experience, device attributes, security, other
Psychological	Self-identity, motivation, affective
Neurological	Neurophysiological, cognitive
Support	Social, environment

Densities were also used to visualize descriptive barrier and facilitator themes and subthemes. For readability, descriptive themes in this section have been indicated by quotation marks, and subthemes have been italicized.

### Major Barriers

Major barrier themes were largely “technological,” more specifically related to user *experience* (24.5% of barrier themes) and *device attributes* (20.1%), and “psychological,” related to *motivation* (15.6%). *User experience* themes summarized in Multimedia Appendix 1 (Table S2) included older adults perceiving themselves as not “tech-savvy” but able to use basic functions such as step count. The perceived obtrusiveness of the PA sensor, both aesthetically and functionally, was a recurrent barrier theme. Devices were perceived to be designed for younger people and not older adults due to form factor, usability, and functionality. A lack of customization and the perceived irrelevance of some metrics hindered sustained engagement. Underlying *device attribute* themes referred to metric accuracy, the ability to register low-intensity activities such as standing, slow walking (strolling), or gardening, and to correctly record other activity types like yoga or strength training. The inability of PA sensors to correctly differentiate between similar activities such as sitting versus standing or to accurately track specific movements relevant to stroke recovery (eg, differentiated movements in hemiparesis) were seen as serious limitations. Users wanted more granular and relevant data (eg, time spent in moderate to vigorous PA) and exercise categorization (eg, fat-burning, cardio, peak); cadence and stride length for running and walking; stairs climbed as measures of vertical movement; swim metrics including laps, stroke type, and count; and strength training metrics such as repetition and set counts. *Motivation* themes referred to the “honeymoon period” or novelty effect of PA sensors quickly diminishing. Users lost interest once they felt they fully understood their activity patterns or had achieved their desired goals. Buzzing reminders were seen as annoying, especially when activity change was not possible, such as while driving. Goals that were not established in partnership with a therapist were often perceived as unrealistic, presenting a significant barrier to adherence. PA sensor unreliability or an inability to accurately measure certain activities (eg, nonlinear occupations and nonrepetitive movements) was demotivating. Users expressed a desire for real-time feedback that helped them to understand the impact of their PA level, for example, on blood sugar, rather than just step counts.

### Major Facilitators

Major facilitator themes were “psychological” related to *motivation* (31.2% of facilitator themes), “technological” related to *user experience* (24.4%), and “support” related to both *social* and *environmental* support (21.4%). Underlying *motivation* themes summarized in Multimedia Appendix 1 (Table S3) included PA sensors that significantly increased self-awareness of PA levels. Achieving goals leads to feelings of inspiration, satisfaction, accountability, and a desire for additional effort. Long-term use was perceived to be strongly linked to intrinsic motivation, driven by personal enjoyment, feelings of accomplishment, and perceived benefits. Features like virtual incentives, nudges, reminders, and other forms of positive encouragement could act as effective extrinsic motivators, especially initially. PA sensors that effectively incorporated behavior change techniques such as self-monitoring, goal setting, and feedback promoted self-efficacy and PA. Individualized and tailored goals, and personalized feedback, for example, based on health status and functional capacity, were seen as critical for sustained motivation. Findings suggested that engagement with PA sensors was often nonlinear, with some users temporarily disengaging and later resuming use rather than abandoning the device entirely. Recognizing these flexible usage patterns is essential for developing interventions that foster long-term adherence. Underlying *user experience* themes included preferences for devices with simple, intuitive interfaces that required minimal user input. Users preferred PA sensors that were comfortable and unobtrusive, which did not interfere with daily activities and routines. Users expressed interest in tracking a more diverse range of activities such as swimming and cycling. The ability to customize goals, feedback type (vibration or visual), frequency of prompts, and data display was critical for effective motivation and sustained use. *Social* support themes related to therapists introducing and setting up the PA sensor, providing clear usage instructions, and interpreting data. This included personalized advice, goal setting, and addressing emotional responses to feedback. Social interaction and group engagement, both face-to-face and online via social networks, were regarded as powerful motivators. Embedding PA sensors into the clinical care process, with therapists actively discussing data and translating it into actionable insights, enhanced the perceived value and utility of the PA sensor. *Self-identity* themes referred to PA sensors significantly enhancing self-awareness of activity levels. Users were often surprised by their baseline level. Objective data helped users clarify real versus perceived activity. Tracking progress and achieving goals, especially with immediate feedback, directly contributed to increased self-efficacy and confidence in the user’s ability to be active and manage their health. For some, using the tracker shifted their self-perception, moving them from a state of pre-contemplation (unmotivated) to maintenance of a healthier lifestyle.

**Figure 2.**
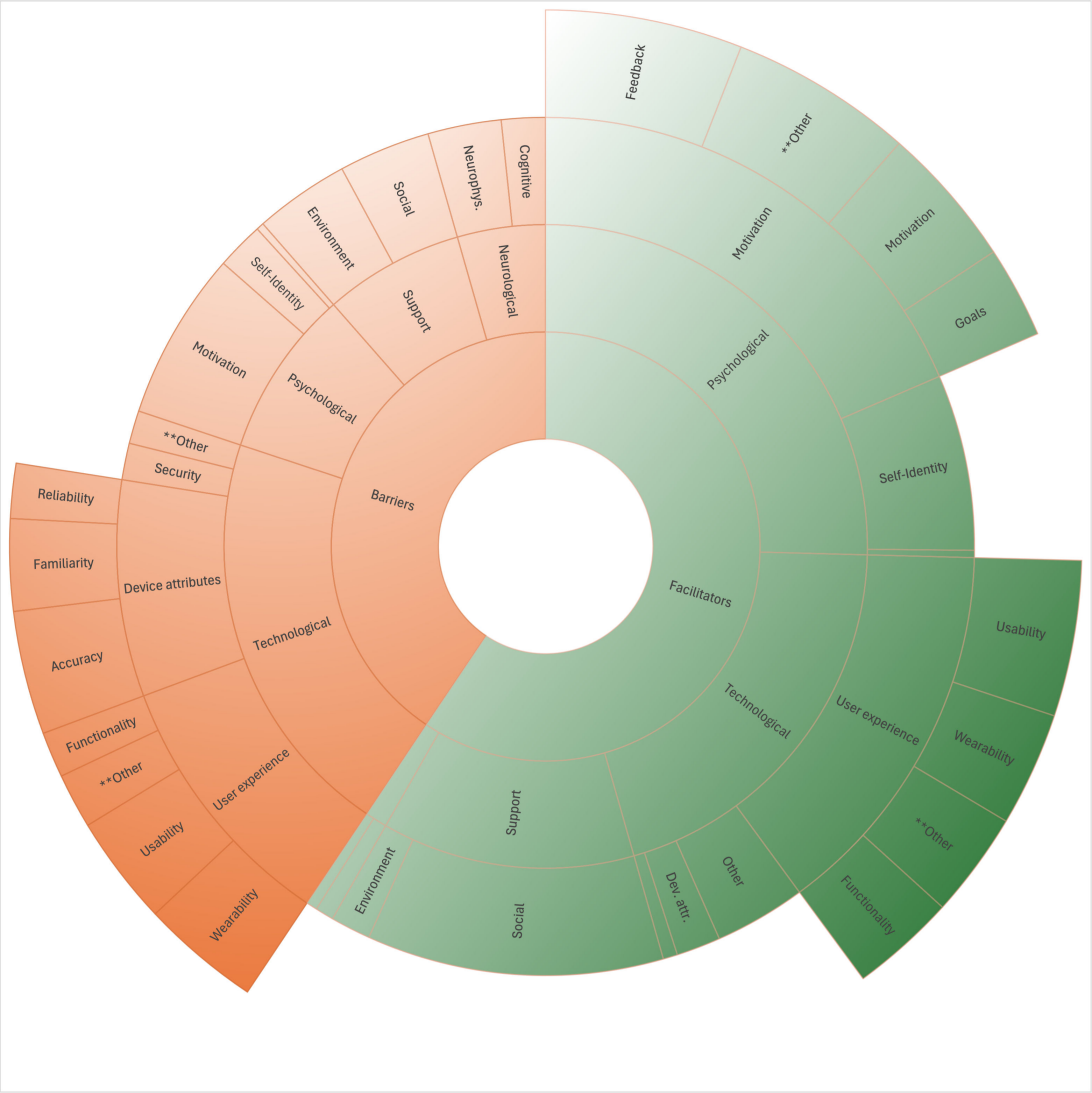
Major barrier/facilitator descriptive and substrate themes. Dev. attr.: device attributes; Neurophys.: neurophysiological

Visualized together in Figure 2, major barriers clustered mainly around *user experience* and *device attribute* aggregates, while facilitators were clustered around *user experience* and *motivation*. Drilling down to the underlying (low-level) themes, it was apparent that major *user experience* barriers were related to *wearability*, *usability*, and *familiarity* in descending order, and major *device attribute* barriers were *accuracy*, *reliability*, and *familiarity* in descending order. For facilitators, which comprised 60% of themes, most were clustered around *motivation*, *user experience*, and *support*. Underlying *motivation*, *feedback* (*motivation* more specifically), and *goals* in descending order were coded most frequently. Major *user experience* descriptive facilitators were *usability*, *wearability*, and *functionality*. Social and environmental *support* facilitators included most notably *professional,* that is, therapist and *caregiver* assistance.

**Figure 3.**
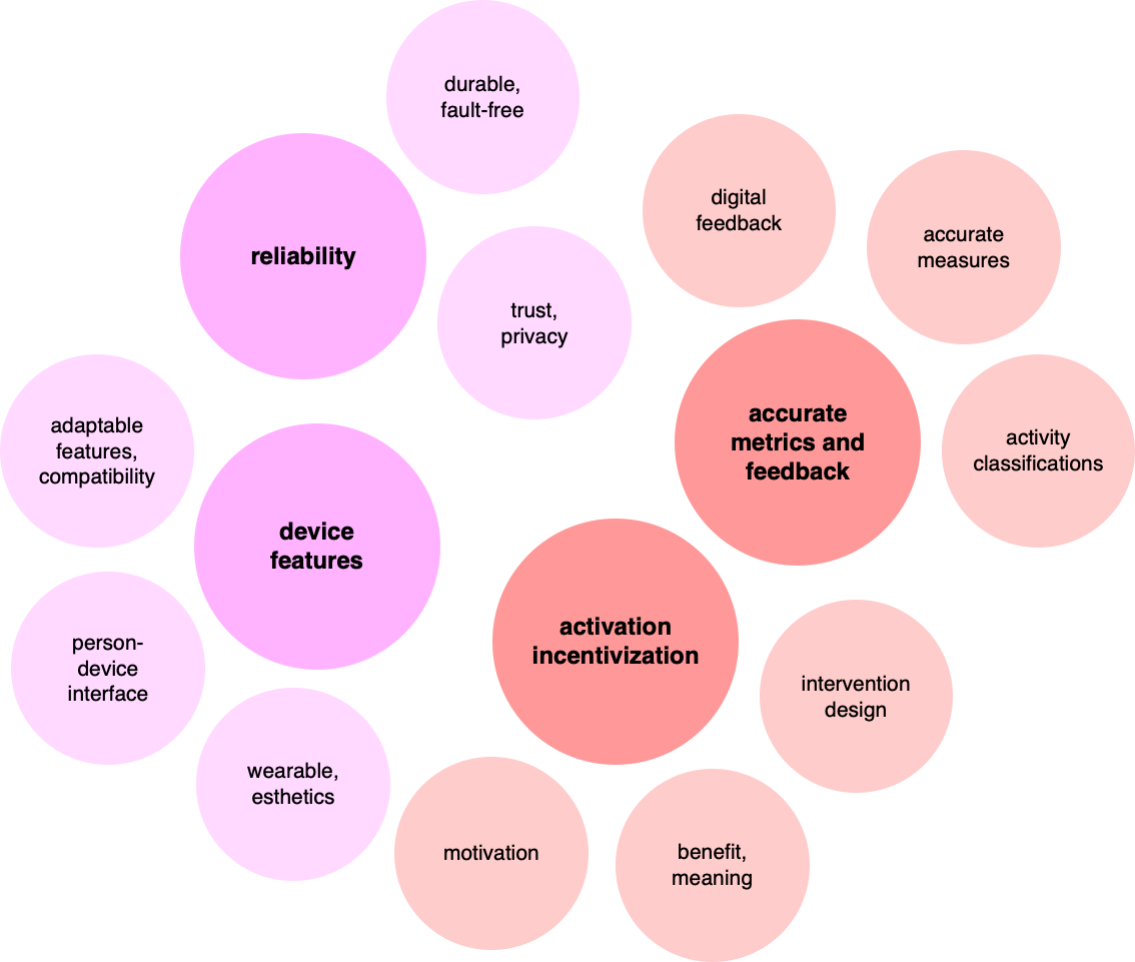
Major barrier clustering by descriptive theme.

The initial analysis revealed major barriers clustered around device themes: subjective *accuracy*, *wearability,* and *usability* (Figure 3). In this context, *accuracy* referred to the perceived precision of the PA sensor; *wearability* to characteristics that influenced the user’s ability and willingness to wear the PA sensor consistently for extended periods (eg, size, fit, esthetics); and *usability* referred to the ease with which users interacted with the PA sensor and companion app.

**Figure 4.**
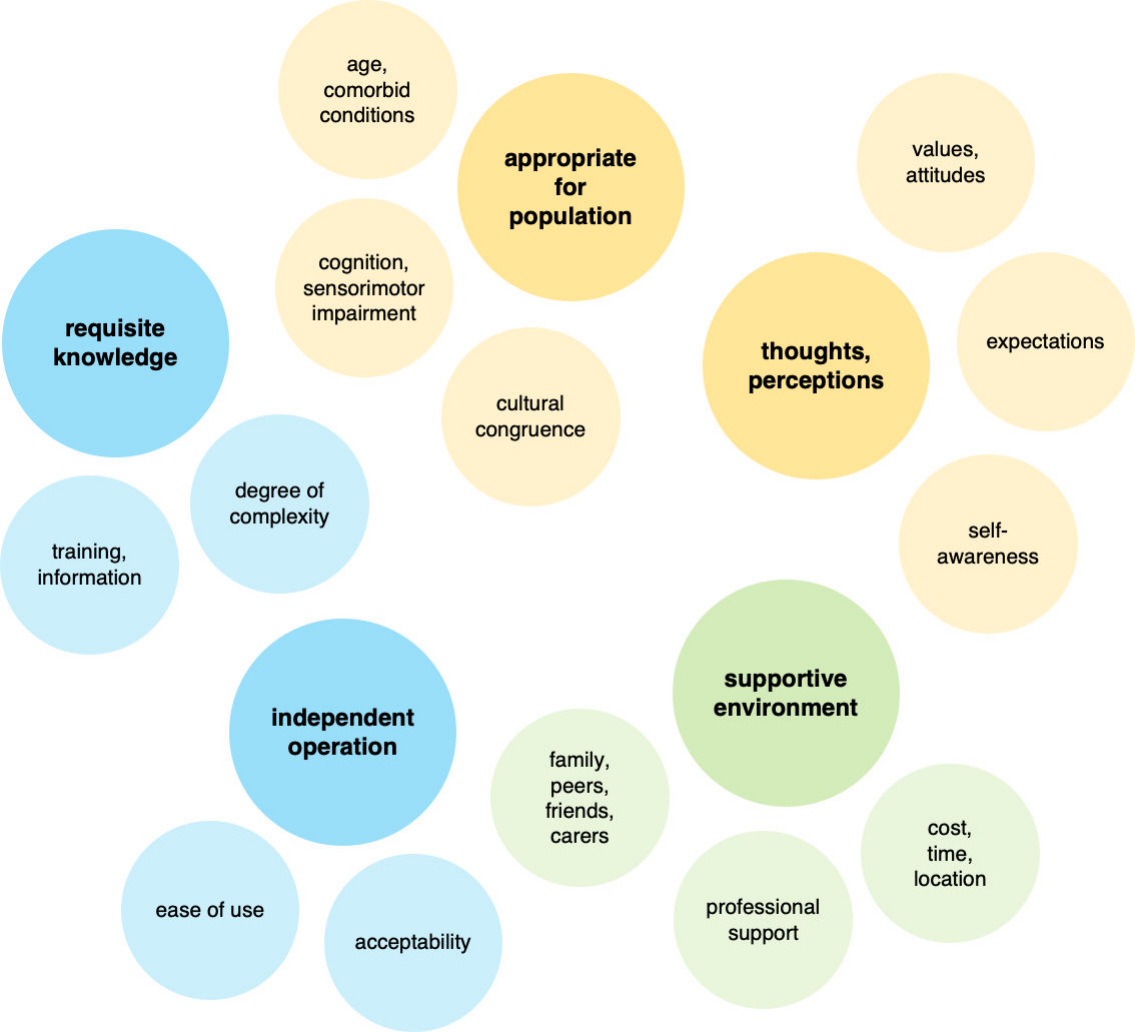
Major facilitator clustering by descriptive theme.

Predominant facilitators were “psychological,” that is, *feedback*, *motivation*, *self-awareness*; device-specific; or related to professional support from the physician or therapist (Figure 4).

**Figure 5.**
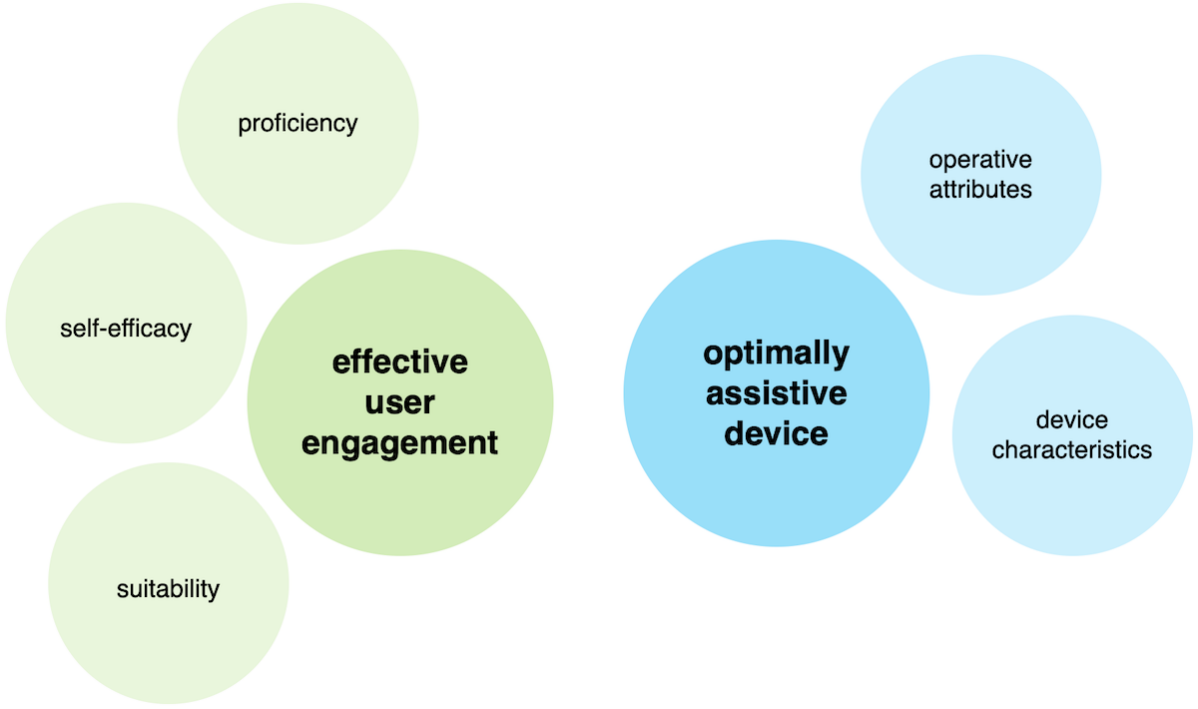
High-level analytical constructs.

Building on the descriptive themes, higher-level analytical or theory-driven constructs were developed. Two distinct analytical constructs emerged, one person-centric, emphasizing factors related to user experience and engagement, and the other essentially device-centric, focused on aspects attributable to the PA sensor itself. Together, these suggested a synergistic interrelationship between effective user engagement and optimally assistive device factors, highlighting their combined contribution to long-term behavior change (Table 4 and Figure 5).

**Table 4.** Analytical constructs, descriptive themes, and subthemes.

Analytical construct and descriptive themes	Subthemes
Effective user engagement	
Proficiency	Requisite skill(s) and knowledge: *degree of complexity; training and information* Independent operation: *acceptability; ease of use*
Self-efficacy	Supportive environment: *cost, commitments, natural environment; professional support; family, peers, friends, carers*
Suitability	Appropriate for population: *cognitive, sensorimotor ability/impairment; cultural congruence; older adults, chronic conditions* Thoughts and preconceptions: *values, attitudes; expectations; self-awareness*
Optimally assistive device	
Operative attributes	Activation, incentivization: *motivation; intervention design; perceived benefit and meaningfulness* Accurate metrics, feedback: *accurate measures; activity type/classification; useful and appropriate feedback*
Device characteristics	Reliability: *durable, fault-free; trust, privacy* Device features: *aesthetics and wearability; person-device interface; adaptable features, compatibility*

Effective user engagement, as a “person-centric” analytical construct, represented the extent to which users meaningfully and effectively interacted with PA sensors. Contributing to this overarching construct were 3 key descriptive themes: *Proficiency* referred to the level of skill and technical knowledge required by the user and their capacity to use the PA sensor independently. Inadequate training, lack of technical skills, inability to resolve usage issues independent of external support, and poor instructions were major technical barrier themes evident in 9 of the studies included in the current analysis [25,26,28,30,32,34-37]. When users felt confident in their ability to operate a PA sensor correctly, they were more likely to engage with it. *Self-efficacy* referred to the individual’s belief in their capacity or readiness to initiate PA to achieve their goals. This encompassed the ability to integrate PA within personal and broader social/cultural contexts to establish or participate in enabling social networks, exercise buddies, peers, and family, and to become better informed about their health, the benefits of regular activity, and maintaining long-term self-motivation. An interaction between motivation and adherence was also evident, suggesting an interplay between the analytical themes discussed in this section, that is, how PA sensors might facilitate a sense of self-efficacy or self-empowerment that in turn promoted increased activity. A lack of self-efficacy or belief in one’s ability to effectively engage in PA and achieve goals was identified as a significant theme in 8 of the studies [22,25,28-30,33,34,39]. Human support from peers, family, coaches, and clinicians was seen as an essential adjunct that aided increased PA together with PA sensor usage; the absence of these supports was evident in 4 studies as major barriers [24,25,32,36]. *Suitability* referred to the appropriateness and acceptability of the PA sensor within the target population. This reflected how well the PA sensor aligned with the physical, cognitive, and cultural needs of the user.

Optimally assistive device, as a “device-centric” analytical construct, referred to the inherent characteristics of the PA sensor and its capacity to effectively promote increased PA. Contributing to this were 2 primary descriptive themes: *Operative attributes* that referred to the functional performance of the PA sensor, accurately capturing and conveying relevant activity data to the user. This included precision, for example, accurate step counts, correct activity classification, and the clarity, relevance, and appropriateness of feedback provided. *Device characteristics,* on the other hand, referred to the design elements of the PA sensor. This included user-friendliness and ease of use of the PA sensor, functional capacity, that is, support for required or expected functionality, timeliness and appropriateness of information provided, and integration with other devices such as the companion app. Perceived inaccuracies and PA sensor defects were major barriers evident in 8 of the studies [23,27,29,33,36,37,40,41]. Similarly, themes that referred to a perceived lack of PA sensor validation, or characteristics that did not appear to have been designed for the abilities of older persons outlined earlier (eg, form factor, usability, functionality), highlighted broader device characteristic issues. Wearability themes reflected how easily people were able to use the PA sensor in everyday life situations and adapt usage to daily routines.

## Discussion

### Principal Findings

This study was primarily undertaken to address noted inconsistencies in the long-term efficacy of device-based PA interventions, particularly in older adults at risk of primary or recurrent stroke. Through a review of the qualitative literature, nuanced human and device-related factors influencing sustained engagement in this specific population were explored through a qualitative lens. By synthesizing lived experiences, insights emerged that explained why these inconsistencies occurred, reflecting individual and contextual factors at play. This qualitative depth is crucial for bridging the implementation gap between technological potential and real-world impact. These findings extend the understanding of existing models by demonstrating how device characteristics must be inherently assistive and person-centric to promote sustained engagement, especially in more vulnerable populations.

The preliminary synthesis was underpinned by a binary schema, categorizing overarching descriptive themes under top-level barrier and facilitator nodes. This implicitly assumed influencing factors were mutually exclusive. However, emergent themes within the included studies suggested a more nuanced reality. A similar observation might be made regarding the conceptual dichotomy between human and device factors. Indeed, one systematic review highlighted an interrelationship between these factors, reporting improved adherence in studies with longer monitoring durations [26]. The authors of the review posited that this could be attributed to increased user confidence over time. Consistent with this finding, studies included in the current analysis similarly indicated an interplay between motivation, technical integrity (eg, absence of defects, data accuracy), and adherence factors, suggesting that these elements are not isolated but rather interact synergistically in complex ways to influence outcomes. Studies included in the current analysis consistently indicated an interplay between motivation and adherence to PA.

Numerous theoretical frameworks have attempted to elucidate behavior change mechanisms through constructs such as self-efficacy (perceived confidence), decisional balance (weighing pros and cons), and processes of change (covert and overt activities facilitating progression) [42]. In the context of PA, self-efficacy specifically refers to an individual’s confidence in identifying and achieving planned PA goals. This confidence is reinforced through mastery experiences, vicarious learning (ie, observing others’ success), persuasion, and performance feedback [40]. While some behavioral goals are characterized by defined end states, others, such as increased PA, necessitate ongoing motivation for sustained maintenance. It has been suggested that individuals are more inclined to adopt healthy behaviors when confronted with a substantial threat and when their actions are perceived as effectively reducing the likelihood or severity of that threat [43]. This applies to patients with stroke concerned about secondary events, as well as individuals at risk of a primary stroke. However, the absence of universally agreed-upon construct measures complicates efforts to isolate which specific intervention components successfully drive behavior change and which do not.

The effectiveness of health care interventions and PA sensors is often complicated by social dynamics, as individuals’ interactions can lead to unforeseen outcomes that alter the intended effects of the intervention [44]. For researchers, deciphering the complex evolution and adaptation of behaviors stemming from these interactions is a formidable task. This complexity is arguably amplified in PA sensor–based interventions, given the intricate interplay of human and device factors. The current study revealed prominent device-specific themes, with technical barriers featuring more significantly than psychological barriers related to motivation, support, adherence, or physiological limitations such as sensorimotor impairment.

While PA sensor–based devices offer objective measures of PA, potentially reducing biases inherent in self-report instruments, themes related to perceived accuracy and trustworthiness were nonetheless evident in this synthesis [28]. The unregulated nature of the consumer market and clinometric quality of consumer-grade PA sensors warrant consideration in this context. A systematic review assessing consumer-grade PA sensors in older adults (mean age 70.2, SD 4.8 y) reported high accuracy for average daily step counts compared to research-grade reference PA sensors [41]. However, the reliability of daily step counts varied considerably across PA sensors, with gait, device placement, and walking speed influencing reliability. Several of the consumer-grade PA sensors contributing to the current thematic synthesis were also evaluated in the aforementioned review. While these findings offer some reassurance regarding accuracy, they simultaneously raise questions about public perceptions of consumer PA sensor accuracy and critical intervention design factors such as training and support. The level of support required for effective PA sensor use also emerged as a recurring major theme in the current study. The degree of intuitiveness and interactivity of the PA sensors may be contributing factors.

The synergistic factors identified in the current study suggest that future interventions should prioritize co-design methodologies, actively involving older patients with stroke in the development and refinement of PA sensors and health care interventions based on these technologies. This would ensure that PA sensors are not only functionally more robust but also intuitively usable, comfortable, and psychologically reinforcing for this specific cohort. For clinicians, these findings underscore the importance of tailored support, including training and goal setting, while addressing individual user concerns regarding PA sensor accuracy and usability when employing these devices in rehabilitation or preventive health care interventions. Future research should focus on developing and rigorously testing co-designed PA sensor–based interventions, employing mixed methods approaches to quantitatively validate the impact of the qualitative insights identified here on sustained levels of PA. Specific areas for investigation might include the optimal frequency and type of professional support, the most effective feedback mechanisms, and ergonomic design features critical in the context of stroke prevention and rehabilitation.

While the hypothesized advantages of PA sensors have garnered considerable scholarly attention, a notable gap exists in the literature regarding their application and, more critically, the lived experience of their use by stroke survivors and older adults at risk of stroke. The current study partially addresses that gap by synthesizing existing qualitative research concerning the utilization of PA sensors within this specific demographic. Through a rigorous qualitative synthesis, both major thematic elements and less predominant, yet illuminating, experiential aspects associated with PA sensor use were identified. The insights gleaned from these findings hold significant implications for the development of more efficacious interventions aimed at mitigating sedentary behavior and fostering increased PA in older adults at risk of primary or recurrent stroke.

### Limitations

Despite a comprehensive search, only 2 included studies specifically addressed older stroke survivors: a qualitative study of 15 survivors and a systematic review incorporating one qualitative stroke study and 5 qualitative proxy population studies [25,26]. While limited stroke-specific data might traditionally constrain the generalizability of these findings, the methodology employed prioritized *conceptual transferability* over statistical representativeness [13]. By extending inclusion to proxy populations with analogous risk and functional profiles, this synthesis identified higher-level analytical constructs that are specifically relevant to older adults at risk of primary or recurrent stroke. These overarching themes provide a robust explanatory framework for PA sensor–based interventions that is theoretically relevant to the stroke experience, even as empirical qualitative data for this population are emerging. However, it is acknowledged that the inherent subjectivity of qualitative interpretation means this synthesis may represent one of several possible frameworks. Furthermore, the lack of detailed technical specifications across the diverse range of included PA sensors precluded an analysis of device-specific effects, such as wearability or discrete usability features. Future research should aim to validate these conceptual constructs through larger, stroke-specific cohorts and more granular investigations into device-specific factors.

### Conclusion

By pooling existing qualitative research, this study contributes a novel and deeper understanding of the benefits and limitations of wearable PA sensors through the subjective experiential lens of older adults at risk of primary or recurrent stroke. This systematic review and thematic synthesis identified a synergistic interplay between human and device-specific factors that influence engagement and sustained increased PA. However, the precise nature of this relationship and its impact on objective PA levels would require verification through higher levels of evidence. These findings provide a framework for more effective PA sensor–based interventions in stroke prevention and rehabilitation, offering valuable insights for technologists designing PA sensors and therapists developing PA sensor–based health interventions for older stroke cohorts.

## Supplementary material

10.2196/86915 Multimedia Appendix 1 Quality and risk of bias assessments for included studies; barrier themes, coded region frequencies (density), and source study counts; facilitator themes, coded region frequencies (density), and study references; and methodological data.

10.2196/86915 Checklist 1 PRISMA 2020 checklist.

## Abbreviations

CASP: Critical Appraisal Skills Program
JBI: Joanna Briggs Institute
PA: physical activity
PRISMA: Preferred Reporting Items for Systematic Reviews and Meta-Analyses
