# Supplementary material for: Patient-Perceived Factors Influencing Physical Activity Sensor Use in Stroke Prevention and Rehabilitation: Systematic Review of Qualitative Studies Using Thematic Synthesis
Source: JMIR Rehabil Assist Technol. 2026 Mar 12;13:e86915. doi: 10.2196/86915 (PMC12981540; doi:10.2196/86915)
Supplement: Multimedia Appendix 1 [file rehab-v13-e86915-s001.docx]

Multimedia Appendix 1: Supplementary Tables and Methodological Data

Manuscript Title: Patient-perceived factors influencing physical activity sensor use in stroke prevention and rehabilitation: a thematic synthesis

Corresponding Author: Paul T. Harris

Table of Contents

[Table S1. Quality and risk of bias assessments for included studies. 3](#_Toc221103062)

[Table S2. Barrier themes, coded region frequencies (density) and source study counts. 4](#_Toc221103063)

[Table S3. Facilitator themes, coded region frequencies (density) and study references. 7](#_Toc221103064)

# Table S1. Quality and risk of bias assessments for included studies.

|  | **JBI Scale Item** | | | | | | | | | |  |
| --- | --- | --- | --- | --- | --- | --- | --- | --- | --- | --- | --- |
| **Author** | **1** | **2** | **3** | **4** | **5** | **6** | **7** | **8** | **9** | **10** | **Overall** |
| Batsis (1) | ⚫ | ⚫ | ⚫ | ⚫ | ⚫ | ⚫ | ⚫ | ⚫ | ⚫ | ⚫ | 🟋🟋 |
| Nguyen (2) | ⚫ | ⚫ | ⚫ | ⚫ | ⚫ | ⚫ | ⚫ | ⚫ | ⚫ | ⚫ | 🟋🟋🟋 |
| Schlomann (3) | ⚫ | ⚫ | ⚫ | ⚫ | ⚫ | ⚫ | ⚫ | ⚫ | ⚫ | ⚫ | 🟋🟋 |
| Hamilton (4) | ⚫ | ⚫ | ⚫ | ⚫ | ⚫ | ⚫ | ⚫ | ⚫ | ⚫ | ⚫ | 🟋🟋 |
| Johansson (5) | ⚫ | ⚫ | ⚫ | ⚫ | ⚫ | ⚫ | ⚫ | ⚫ | ⚫ | ⚫ | 🟋🟋 |
| Farina (6) | ⚫ | ⚫ | ⚫ | ⚫ | ⚫ | ⚫ | ⚫ | ⚫ | ⚫ | ⚫ | 🟋🟋🟋 |
| Kononova (7) | ⚫ | ⚫ | ⚫ | ⚫ | ⚫ | ⚫ | ⚫ | ⚫ | ⚫ | ⚫ | 🟋🟋 |
| Western (8) | ⚫ | ⚫ | ⚫ | ⚫ | ⚫ | ⚫ | ⚫ | ⚫ | ⚫ | ⚫ | 🟋🟋 |
| Whelan (9) | ⚫ | ⚫ | ⚫ | ⚫ | ⚫ | ⚫ | ⚫ | ⚫ | ⚫ | ⚫ | 🟋🟋 |
| Stove (10) | ⚫ | ⚫ | ⚫ | ⚫ | ⚫ | ⚫ | ⚫ | ⚫ | ⚫ | ⚫ | 🟋🟋🟋 |
| Ummels (11) | ⚫ | ⚫ | ⚫ | ⚫ | ⚫ | ⚫ | ⚫ | ⚫ | ⚫ | ⚫ | 🟋🟋 |
| Ostlind (12) | ⚫ | ⚫ | ⚫ | ⚫ | ⚫ | ⚫ | ⚫ | ⚫ | ⚫ | ⚫ | 🟋🟋🟋 |
| Gualtieri (13) | ⚫ | ⚫ | ⚫ | ⚫ | ⚫ | ⚫ | ⚫ | ⚫ | ⚫ | ⚫ | 🟋🟋 |
| Mercer (14) | ⚫ | ⚫ | ⚫ | ⚫ | ⚫ | ⚫ | ⚫ | ⚫ | ⚫ | ⚫ | 🟋🟋🟋 |
| Randriambelonoro (15) | ⚫ | ⚫ | ⚫ | ⚫ | ⚫ | ⚫ | ⚫ | ⚫ | ⚫ | ⚫ | 🟋🟋 |
| Ehn (16) | ⚫ | ⚫ | ⚫ | ⚫ | ⚫ | ⚫ | ⚫ | ⚫ | ⚫ | ⚫ | 🟋🟋🟋 |
| Takemoto (17) | ⚫ | ⚫ | ⚫ | ⚫ | ⚫ | ⚫ | ⚫ | ⚫ | ⚫ | ⚫ | 🟋🟋 |
| Brickwood (18) | ⚫ | ⚫ | ⚫ | ⚫ | ⚫ | ⚫ | ⚫ | ⚫ | ⚫ | ⚫ | 🟋🟋🟋 |

# Table S2. Barrier themes, coded region frequencies (density) and source study counts.

| Theme | Subtheme | Density | Description | Underlying concepts | References |
| --- | --- | --- | --- | --- | --- |
| Technological | User Experience  - customisability  - functionality  - usability - usefulness - wearability | 72 (15) | Holistic perception and emotional responses users have while interacting with PA sensors within their recovery context. This included usability (ease of use, intuitive design), usefulness (perceived helpfulness), and wearability (how appealing or motivating). | - Technical proficiency and setup challenges - Limited feature engagement - Physical discomfort and annoyance - Device aesthetics and integration into daily routine - Age-related design and accessibility issues - Battery life and charging burden - Perceived usefulness and lack of customisation | Brickwood (18)  Ehn (16)  Farina (6)  Gualtieri (13)  Hamilton (4)  Johansson (5)  Kononova (7)  Mercer (14)  Nguyen (2)  Randriambelonoro (15)  Schlomann (3)  Stove (10)  Takemoto (17)  Ummels (11)  Whelan (9) |
|  | Device Attributes - accuracy - familiarity - reliability | 59 (15) | Inherent characteristics and functionalities of the PA sensor itself that influence an older stroke patient’s experience and engagement, shaping how the PA sensor is perceived, used, and integrated into daily life and recovery. | - Accuracy and validity of the data - Reliability and technical functionality - Complexity and usability - Instructional support and language - measurement specificity - Technical integration and compatibility issues - Influence on motivation and engagement | Brickwood (18)  Ehn (16)  Farina (6)  Gualtieri (13)  Hamilton (4)  Johansson (5)  Kononova (7)  Mercer (14)  Nguyen (2)  Ostlind (12)  Randriambelonoro (15)  Stove (10)  Takemoto (17)  Ummels (11)  Whelan (9) |
|  | *Security* - damage - privacy - safety | 10 (6) | Related to the confidentiality, integrity, and availability of data collected, stored, and transmitted by PA sensors, as well as the vulnerability of the PA sensor itself to unauthorised use. | - Data confidentiality and sharing concerns - Privacy awareness and understanding of associated risks - Data integrity and control - Physical security and durability | Brickwood (18)  Ehn (16)  Mercer (14)  Randriambelonoro (15)  Ummels (11)  Whelan (9) |
| Psychological | Motivation - accountability - adherence - attainment - feedback - goals - habits - motivation - spiritual | 46 (15) | Intrinsic and extrinsic factors drivers that compel the older stroke patient to initiate and sustain engagement in physical activity, mediated by the feedback from the PA sensor. In this context intrinsic factors may be desire for good health and well-being, while and extrinsic factors could be goals set by therapist or the individual. | - Initial novelty vs sustained engagement - Perceived lack of value - Irrelevant and/or unhelpful features and feedback - Desire for actionable and contextual feedback - Negative emotional response(s) to feedback - Device focus and user goals mismatch - Unrealistic or unachievable goals - Impact of technical issues on motivation - Interruption of routine(s) - Requirement for baseline motivation | Batsis (1)  Brickwood (18)  Ehn (16)  Farina (6)  Gualtieri (13)  Hamilton (4)  Kononova (7)  Nguyen (2)  Ostlind (12)  Randriambelonoro (15)  Schlomann (3)  Takemoto (17)  Ummels (11)  Western (8)  Whelan (9) |
|  | *Self-Identity* - autonomy - competence - preconceptions - self-awareness - self-efficacy | 13 (8) | The stroke patient’s evolving sense of self, influenced by their experience and interaction with the PA sensor. This might include shifts from pre-stroke identities, the integration of a "patient" identity and how engagement with physical activity by using a PA sensor reshapes perception of the individual’s capabilities and overall self in the recovery journey. | - Pre-existing perceptions of technology - Negative perceptions of PA sensor(s) - Established routines and perceived self-efficacy - Impact of PA confidence/activation - Underestimation of inactivity - Scepticism and overwhelm - Low self-esteem | Batsis (1)  Brickwood (18)  Gualtieri (13)  Hamilton (4)  Kononova (7)  Ostlind (12)  Western (8)  Whelan (9) |
| Support | *Environment* - safety - terrain - weather - attitudes - commitments - cost - cultural - norms - strata | 26 (10) | The natural and structural i.e. built characteristics of an older stroke patient's surroundings that either enable or impede engagement in physical activity, particularly as it relates to the use and utility of PA sensors e.g., weather, safe pathways, ramps, rural/city, etc. | - Financial cost - Time constraints and competing obligations/commitments - Perceived stigmatisation and self-consciousness - Lack of accessibility and ability of PA sensors - Healthcare system limitations - Influence of weather and climate - Dislike of traditional exercise environments - Geographic/mobility limitations | Batsis (1)  Brickwood (18)  Gualtieri (13)  Kononova (7)  Johansson (5)  Mercer (14)  Randriambelonoro (15)  Schlomann (3)  Ummels (11)  Western (8) |
|  | *Social* - caregiver - information - peer - professional - training | 25 (12) | The perceived and actual resources, assistance, and positive interactions exchanged within a stroke survivor's interpersonal network, which significantly influence their engagement in physical activity and their experience with PA sensors. This construct encompasses various forms of aid, including peer, caregiver, family, and particularly professional support. | - Limited organic social interaction - Need for structured support - Professional support gaps and challenges - Insufficient therapist training - Prioritisation treatment of more severe deficits - Resource intensity - Language and accessibility of information - Impatient or frustrated informal support - External motivation vs. sustained behaviour change - Perceived “otherness” of technology | Ehn (16)  Gualtieri (13)  Hamilton (4)  Johansson (5)  Kononova (7)  Mercer (14)  Randriambelonoro (15)  Schlomann (3)  Stove (10)  Ummels (11)  Western (8)  Whelan (9) |
| Neurological | *Neurophysiological* - balance - gait - motor - sensory - vision - endurance - fatigue - flexibility - injury - mobility - strength | 20 (12) | Brain and body impacts of stroke such as balance, gait and sensory abilities, and how these interact with physical activity and the use of PA sensors. | - Fine and gross motor impairments - Cognitive impairments - Sensory perception deficits - Gait and balance impairments - Impact of comorbidities and health status - Reduced walking speed, and mobility limitations - Physical incapacity to increase activity levels | Batsis (1)  Brickwood (18)  Ehn (16)  Gualtieri (13)  Hamilton (4)  Johansson (5)  Kononova (7)  Mercer (14)  Randriambelonoro (15)  Schlomann (3)  Stove (10)  Western (8) |
|  | *Cognitive* - attention - awareness - comprehension - language - memory - planning | 12 (7) | The older stroke patient’s mental processes and cognitive functions that influence engagement with physical activity and interaction with PA sensors. | - Memory impairments - Cognitive load and demands - Problem-solving and understanding - Attention and executive function - Severity of cognitive impairment - Emotional and cognitive response(s) to feedback | Gualtieri (13)  Hamilton (4)  Mercer (14)  Randriambelonoro (15)  Schlomann (3)  Stove (10)  Western (8) |

Note: underscore = major theme, *italics* = minor theme.

# Table S3. Facilitator themes, coded region frequencies (density) and study references.

| Theme | Subtheme | Density | Description | Underlying concepts from studies | References |
| --- | --- | --- | --- | --- | --- |
| Psychological | Motivation - accountability - adherence - attainment - feedback - goals - habits - motivation - spiritual | 134 (18) | Internal and external drivers that compel stroke survivors to initiate and sustain engagement in physical activity, often mediated by the feedback from a PA sensor. This encompasses both intrinsic (e.g. desire for good health and well-being) and extrinsic factors e.g. goals set by therapist and self, and the crucial element of self-efficacy, or a survivor's belief in their ability to perform activities despite challenges. | - Self-awareness and feedback - Goal setting and achievement - intrinsic motivation and autonomy - extrinsic motivational factors - behaviour change techniques - integration into daily routines/habits - personalization and adaptability - social relatedness - emotional impact(s) - persistent engagement | Batsis (1)  Brickwood (18)  Ehn (16)  Farina (6)  Gualtieri (13)  Hamilton (4)  Johansson (5)  Kononova (7)  Mercer (14)  Nguyen (2)  Ostlind (12)  Randriambelonoro (15)  Schlomann (3)  Stove (10)  Takemoto (17)  Ummels (11)  Western (8) Whelan (9) |
|  | Self-Identity - autonomy - competence - preconceptions - self-awareness - self-efficacy | 48 (14) | The stroke patient’s evolving sense of self, influenced by their experience and interaction with the PA sensor. This might include shifts from pre-stroke identities, the integration of a "patient" identity and how engagement with physical activity reshapes perception of the individual’s capabilities and overall self in the recovery journey. | - Increased self-awareness - Boost in self-efficacy and confidence - Empowerment and self-management - Shift in identity perception - Sense of accomplishment and pride - Health-related identity - Personalized “game” and self-competition - Curiosity and discovery | Brickwood (18)  Ehn (16)  Farina (6)  Gualtieri (13)  Hamilton (4)  Kononova (7)  Mercer (14)  Nguyen (2)  Ostlind (12)  Randriambelonoro (15)  Schlomann (3)  Takemoto (17)  Ummels (11)  Western (8) |
| Technological | User Experience - customisability  - functionality  - usability - usefulness - wearability | 105 (16) | Holistic perception and emotional responses users have while interacting with PA sensors within their recovery context, including usability (ease of use, intuitive design), usefulness (perceived helpfulness), and wearability (how appealing or motivating). User experience related to perceived usability, usefulness, wearability, functionality, and customisability. | - Simplicity and ease of use - Wearability and comfort - Readability and optimal display - Practical functionality and features - Useful, actionable feedback - Customisation and personalization - Perceived usefulness and relevance to health concerns - Effective motivational feedback and prompts - Positive impact on PA | Brickwood (18)  Ehn (16)  Farina (6)  Gualtieri (13)  Hamilton (4)  Johansson (5)  Kononova (7)  Mercer (14)  Nguyen (2)  Ostlind (12)  Randriambelonoro (15)  Schlomann (3)  Stove (10)  Takemoto (17)  Ummels (11) Whelan (9) |
|  | *Device Attributes* - accuracy - familiarity - reliability | 21 (11) | Inherent characteristics and functionalities of the PA sensor itself that influence an older stroke patient’s experience and engagement, shaping how the PA sensor is perceived, used, and integrated into daily life and recovery. | - Accuracy and validity of measurements - Reliability and consistency - Targeted measurement capabilities - Long battery life - Objective data for subjective limitations - Essential features and functionalities - Perceived usefulness and meaningfulness | Brickwood (18)  Ehn (16)  Farina (6)  Gualtieri (13)  Johansson (5)  Kononova (7)  Randriambelonoro (15)  Schlomann (3)  Takemoto (17) Ummels (11) Western (8) |
| Support | Social - caregiver - information - peer - professional - training | 81 (14) | Refers to perceived and actual provision of resources and assistance from an individual's social network, contributing to their well-being and ability to cope with life challenges. This encompasses emotional support (e.g., empathy, encouragement), informational support (e.g., advice, guidance), tangible or instrumental support (e.g., practical aid, material assistance), and appraisal support (e.g., affirmation, feedback that aids self-evaluation). | - Professional guidance and support - Tailored and individualised support - Peer and group support - Family and care-giver involvement - Accountability and external monitoring - Provision of information and education - Seamless integration into care - Positive social influence | Batsis (1)  Brickwood (18)  Ehn (16)  Farina (6)  Gualtieri (13)  Hamilton (4)  Kononova (7)  Mercer (14)  Nguyen (2)  Ostlind (12)  Randriambelonoro (15)  Schlomann (3)  Ummels (11) Western (8) |
|  | *Environment* - safety - terrain - weather - attitudes - commitments - cost - cultural - norms - strata | 11 (7) | Refers to the physical and structural characteristics of an older stroke survivor's surroundings that either facilitate or impede physical activity. This includes accessibility of their home and community (e.g., ramps, clear pathways), the availability of safe and spaces for exercise (e.g., parks, accessible gyms), and the presence of technologies or resources (e.g. PA sensor and exercise equipment) that make physical activity more feasible. It also encompasses ambient factors like weather, lighting, noise, and safety. | - Affordability and accessibility of PA sensors - Cost-effectiveness of intervention delivery - Positive perception of external factors - Availability of PA sensors recommended by healthcare professionals - Perceived freedom and independence - Validation with normative data | Brickwood (18)  Gualtieri (13)  Kononova (7)  Nguyen (2)  Ostlind (12)  Schlomann (3) Western (8) |

Note: underscore = major theme, *italics* = minor theme.

**References**

1. Batsis JA, Naslund JA, Gill LE, Masutani RK, Agarwal N, Bartels SJ. Use of a wearable activity device in rural older obese adults: A pilot study. Gerontology and Geriatric Medicine. 2016;2.

2. Nguyen NH, Hadgraft NT, Moore MM, Rosenberg DE, Lynch C, Reeves MM, et al. A qualitative evaluation of breast cancer survivors' acceptance of and preferences for consumer wearable technology activity trackers. Support Care Cancer. 2017;25(11):3375–84.

3. Schlomann A. A case study on older adults' long-term use of an activity tracker. Gerontechnology. 2017;16(2):115–24.

4. Hamilton C, McCluskey A, Hassett L, Killington M, Lovarini M. Patient and therapist experiences of using affordable feedback-based technology in rehabilitation: a qualitative study nested in a randomized controlled trial. Clinical Rehabilitation. 2018;32(9):1258–70.

5. Johansson D, Malmgren K, Alt Murphy M. Wearable sensors for clinical applications in epilepsy, Parkinson's disease, and stroke: a mixed-methods systematic review. J Neurol. 2018;265(8):1740–52.

6. Farina N, Sherlock G, Thomas S, Lowry RG, Banerjee S. Acceptability and feasibility of wearing activity monitors in community-dwelling older adults with dementia. Int J Geriatr Psychiatry. 2019;34(4):617–24.

7. Kononova A, Li L, Kamp K, Bowen M, Rikard RV, Cotten S, et al. The Use of Wearable Activity Trackers Among Older Adults: Focus Group Study of Tracker Perceptions, Motivators, and Barriers in the Maintenance Stage of Behavior Change. JMIR Mhealth And Uhealth. 2019;7(4):e9832–e.

8. Western MJ, Thompson D, Peacock OJ, Stathi A. The impact of multidimensional physical activity feedback on healthcare practitioners and patients. BJGP open. 2019;3(1).

9. Whelan ME, Orme MW, Kingsnorth AP, Sherar LB, Denton FL, Esliger DW. Examining the Use of Glucose and Physical Activity Self-Monitoring Technologies in Individuals at Moderate to High Risk of Developing Type 2 Diabetes: Randomized Trial. JMIR Mhealth Uhealth. 2019;7(10):e14195.

10. Stove MP, Larsen BT. Self-monitoring-usability evaluation of heart rate monitoring using wearable devices in patients with acquired brain injury. European Journal of Physiotherapy. 2019.

11. Ummels D, Beekman E, Moser A, Braun SM, Beurskens AJ. Patients' experiences with commercially available activity trackers embedded in physiotherapy treatment: a qualitative study. Disabil Rehabil. 2019:1–9.

12. Östlind E, Ekvall Hansson E, Eek F, Stigmar K. Experiences of activity monitoring and perceptions of digital support among working individuals with hip and knee osteoarthritis – a focus group study. BMC Public Health. 2022;22(1):1641.

13. Gualtieri L, Rosenbluth S, Phillips J. Can a Free Wearable Activity Tracker Change Behavior? The Impact of Trackers on Adults in a Physician-Led Wellness Group. JMIR Res Protoc. 2016;5(4):e237.

14. Mercer K, Giangregorio L, Schneider E, Chilana P, Li M, Grindrod K. Acceptance of Commercially Available Wearable Activity Trackers Among Adults Aged Over 50 and With Chronic Illness: A Mixed-Methods Evaluation. 2016. p. 168–84.

15. Randriambelonoro M, Chen Y, Geissbuhler A, Pu P, editors. Exploring physical activity monitoring devices for diabetic and obese patients. UbiComp and ISWC 2015 - Proceedings of the 2015 ACM International Joint Conference on Pervasive and Ubiquitous Computing and the Proceedings of the 2015 ACM International Symposium on Wearable Computers; 2015.

16. Ehn M, Eriksson LC, Akerberg N, Johansson AC. Activity Monitors as Support for Older Persons' Physical Activity in Daily Life: Qualitative Study of the Users' Experiences. JMIR MHealth and UHealth. 2018;6(2):e34.

17. Takemoto M, Lewars B, Hurst S, Crist K, Nebeker C, Madanat H, et al. Participants' Perceptions on the Use of Wearable Devices to Reduce Sitting Time: Qualitative Analysis. JMIR MHealth and UHealth. 2018;6(3):e73.

18. Brickwood KA-O, Williams AD, Watson G, O'Brien J. Older adults' experiences of using a wearable activity tracker with health professional feedback over a 12-month randomised controlled trial. (2055-2076 (Print)).
